# Comparative Study of *Bacillus amyloliquefaciens* X030 on the Intestinal Flora and Antibacterial Activity Against *Aeromonas* of Grass Carp

**DOI:** 10.3389/fcimb.2022.815436

**Published:** 2022-01-25

**Authors:** Pengji Zhou, Wenhui Chen, Zirong Zhu, Kexuan Zhou, Sisi Luo, Shengbiao Hu, Liqiu Xia, Xuezhi Ding

**Affiliations:** State Key Laboratory of Developmental Biology of Freshwater Fish, Hunan Provincial Key Laboratory of Microbial Molecular Biology, College of Life Science, Hunan Normal University, Changsha, China

**Keywords:** *Bacillus amyloliquefaciens*, aquaculture disease, proteomics, intestinal flora, grass carp

## Abstract

Beneficial microorganisms to control bacterial diseases has been widely used in aquaculture, *Bacillus amyloliquefaciens* (BaX030) as a probiotic feed additive was a commonly biological control method. Added sucrose promoted the growth of BaX030, and the yield of its antibacterial substance macrolactin A was enhanced by 1.46-fold. A total of 2055 proteins were screened through proteomics, with 143 upregulated and 307 downregulated. Differential protein expression analysis and qRT-PCR verification showed that the pentose phosphate pathway and the fatty acid synthesis pathway were upregulated, thereby providing sufficient energy and precursors for the synthesis of macrolactin A. The influence of some potential regulatory factors (SecG, LiaI, MecG and ComG) on macrolactin A was discovered. After grass carp were fed with BaX030, the abundance of probiotics (*Fusobacterium*, *Proteobacteria*, *Gemmobacter*) were higher than the control group, and the abundance of potential pathogenic bacteria (*Planctomycetes*, *Aeromonas*) were significantly lower than the control group. The cell and challenge experiments showed that BaX030 can significantly increase the expression of *C3* and *IL8* in the liver and kidney, which decreases the risk of immune organ disease. Moreover, BaX030 effectively reduced the mortality of grass carp. The results revealed that BaX030 can significantly improve the structure of the intestinal flora, enhance immunity and it is beneficial to the control of grass carp *Aeromonas*.

## Highlights

- BaX030 changed the intestinal flora, including *Fusobacterium*, *Proteobacteria* and *Planctomycetes*
- Sucrose affected the growth and the macrolactin A synthesis of BaX030
- BaX030 can improve the specific immunity in grass carp

## Introduction

The increasing density of fish farming and the deterioration of the aquatic environment have led to disease outbreaks and aquaculture losses. The traditional extensive use of antibiotics will lead to environmental pollution, pathogen resistance and drug residues in aquatic products, which can seriously threaten human health (Mukherjee et al., 2019; Zhi et al., 2020). Therefore, improving the microbial community by probiotic supplementation has been suggested as promising approach in the prevention of diseases. *Bacillus amyloliquefaciens* can produce a large number of secondary metabolites with a wide range of antibacterial activities, including polyketide compounds (bacillaene, macrolactin and difficidin) and lipopeptide compounds (surfactins, iturins and fengycins) (Debois et al., 2014; Cochrane and Vederas, 2016). As an effective strategy of biological control, it has great potential in aquaculture.

At present, systematically mining multiple core genes and proteins has become a research trend; it explains the molecular mechanism underlying the synthesis of secondary metabolites and the increase of their production through genomics combined with transcriptomics, metabolomics or proteomics (Chavali and Rhee, 2018; Xu et al., 2018; Zhao et al., 2018). Proteomics has produced a variety of novel leads for the analysis of protein traffic in *Bacillus*. These studies proved that the availability of genomics, proteomics and bioinformatics tools can effectively increase the yield of secondary metabolites and modify related gene clusters.

The intestine of grass carp is the organ with the largest and the most complex microbial flora. The gut microbiota is colonized by diverse probiotics and pathogenic microbes (Pérez et al., 2010; Zhou et al., 2019), and it is involved in many physiological processes of the host and plays crucial roles, such as enhancing host metabolism, nutrient absorption, growth and disease resistance. (Rooks and Garrett, 2016; Blander et al., 2017; Huang et al., 2018; Rimoldi et al., 2018); therefore, it has been widely studied worldwide. However, the intestine can also become an important entry point for bacteria to invade grass carp. Under artificial breeding conditions, grass carp are easily infected with bacterial diseases and die, which causes huge economic losses (Song et al., 2014). For example, bacterial sepsis is caused by *Aeromonas hydrophila* (Elbehiry et al., 2019), skin ulcers, and severe ascites are caused by *Aeromonas veronii* (Li et al., 2020), and these species often colonize the intestine to exert pathogenicity. Enteric inflammation is another important factor that leads to the high mortality of grass carp (Tran et al., 2018) because of the disorder it causes to the structure, function, and metabolism of the microbial flora. Carding et al. (2015) found that enteric pathogens trigger both local and systemic inflammation that changes the composition of microbiota and barrier functions. Therefore, studying the intestinal microbiota and probiotics has great significance for the prevention and treatment of intestinal diseases. The latest third-generation sequencing (TGS) is characterized by single molecules and long reads, which may enable high-throughput full-length 16S sequencing. TGS has reached a level of species identification accuracy similar to that of first-generation sequencing and the breadth of application similar to that of the next-generation sequencing (NGS), and it is expected to be widely used in the study of intestinal flora in the future (Van Dijk et al., 2018; Kumar et al., 2019).

We discovered the effect of sucrose on bacterial growth and macrolactin A production. *B.amyloliquefaciens* X030 (BaX030) provided immune protection to grass carp against infection by *A. hydrophila* and *A. veronii in vivo* and *in vitro*. Differences in the intestinal microbiota between diseased and healthy grass carp were investigated by TGS technology. Our results are important in the prevention and treatment of intestinal diseases by controlling gut microbes.

## Materials and Methods

### Bacterial Strains and Culture Conditions

BaX030 was screened and isolated from soil in Henan Province, China. It is currently deposited in the Wuhan Center for Culture Collection (CCTCC NO: M2014159), and the whole-genome sequence data are available in the GenBank database under the accession number CP040672.1. Cao et al. (2019) and Huang et al. (2020) isolated *A. hydrophila* X040 (AhX040) (GenBank: KU159281.1) and *A. veronii* X005 (AvX005) (GenBank: KU641116.1) from diseased grass carp on a Wangcheng fish farm (Changsha, Hunan Province, China), respectively. They were common bacteria in freshwater, sewage, silt and soil, and ther were also the main pathogenic bacteria that caused diseases in freshwater farmed. The LB medium contained 10 g/L NaCl, 10 g/L tryptone, and 5 g/L yeast extract. The fermentation medium consisted of LB medium supplemented with 32 g/L sucrose.

After BaX030 was activated overnight, it was transferred to LB medium or fermentation medium at a ratio of 3% and named CG+BaX030 and SG+BaX030, respectively. Three replicates were performed for each group. The fermentation broth was diluted to a certain concentration every 4 h, and the OD_600nm_ value was measured with a SmartSpecTM 3000 spectrophotometer (Bio-Rad, Berkeley, USA). A growth curve was drawn with the culture time at the abscissa and the OD_600nm_ value at the ordinate. Small amounts of fermentation broth were taken at different time points during growth, centrifuged at 10000 rpm for 2 min to collect the bacteria. An AXIO Observer A1 upright optical microscope (Zeiss, Jena, Germany) was used for observation.

### Separation and Purification of Antibacterial Active Substances

BaX030 was cultured on the LB medium and the fermentation medium for 48 h, and its fermentation broth was centrifuged at 10000 rpm for 10 min. The supernatant was added to an equal volume of ethyl acetate overnight extraction, and the upper organic phase was freeze-dried, dissolved in 100% methanol and analysed by high-performance liquid chromatography (HPLC 1290, Agilent, Palo Alto, USA). The injection volume was 10 µL, the column was a reversed-phase ZORBAX SB-C18 column (4.6×150 mm, 5.0 µm), and the flow rate was 1 mL/min, with mobile phases A consisting of 10% acetonitrile (ACN) and B consisting of 90% ACN. Mobile phase B was gradient eluted from 100% (0 min) to 0% (20 min), and the detection wavelength was 280 nm. After collecting and freeze-drying the elution peaks, antibacterial experiments were used to detect the activity of the peak by the filter paper method with AvX005 and AhX040 as indicator bacteria. The activity peak was polyketide macrolactin A (Zhou et al., 2021). The concentration of macrolactin A (the standard curve was drawn after quantification by HPLC 1290) was determined using the following formula: macrolactin A: y=1801.1x+1.23 (R^2 =^ 0.9989) where y is the peak area and x is the macrolactin A concentration.

### Whole-Cell Protein Extraction and SDS-PAGE

We extracted the whole protein of BaX030 at 20 h, the protein concentration was determined by a quantitative proteomics kit (Sangon Biotech, Shanghai, China) and microplate reader (Spectral Max M5, Silicon Valley, USA). The microplate reader was used to measure the absorbance at 595 nm, and the protein concentration in the sample was calculated according to the standard curve. Both BSA standards and samples were repeated twice. SDS-PAGE detected whether the protein was degraded, the band analysis was performed with Gel-Pro Analyzer 4.0 software. The samples were finally sent to Shanghai Medical College of Fudan University for isobaric tags for relative and absolute quantification (iTRAQ).

### Proteolysis and iTRAQ

One tube of iTRAQ reagent was added per 100 µg peptide, incubated at room temperature for 2 h, mixed with 50 µL ultrapure water and left at room temperature for 30 min; each group of labelled products was combined in one tube and then dried by vacuum concentrator. The peptide samples were reconstituted in loading buffer of ultra performance liquid chromatography (UPLC), and it was performed on a Waters ACQUITY UPLC by using a reversed-phase C18 column (1.7 µm, 3 mm × 150 mm, Waters, Milford, USA) (Bekker-Jensen et al., 2017; Hu et al., 2019). According to the BaX030 genome annotation, the SwissProt/UniProt database was used for protein identification. The critical range of fold change (FC) < 0.667 and FC > 1.50 was used to screen different proteins (*P* < 0.05). Data are available *via* ProteomeXchange with identifier PXD024498 (Reviewer account details: Username: reviewer_pxd024498@ ebi.ac.uk; Password: etGXrDFP).

### BaX030 on the Immune Protection of Grass Carp

We took 120 healthy grass carp (weight 4.6 ± 2.0 g, body length 6.5 ± 1.0 cm), and kept them in 30 L fish tanks, the water volume of each fish tank was 20 L. This was two groups (fed without BaX030, control group, and fed with BaX030, experimental group) in triplicates. BaX030 was counted by the plate colony counting method, and the amount of bacteria mixed into the feed was 1×10^9^ colony forming units/gram (CFU/g). It was cultured in fermentation medium for 20 h, 1.5 mL of bacterial liquid was mixed with 1 g of feed, and the bacterial content of the feed was 1×10^9^ CFU/g. The daily feed amount was 3% of the fish’s weight given twice a day. After all fish were fed for 30 days, 3 fish were randomly taken from the WT group and the BA group, their intestinal contents were collected for TGS. We took 6 other fish and intraperitoneally injected them with 0.1 mL of 1×10^6^ colony forming units/milliliter (CFU/mL) AhX040 or 0.15 mL of 1×10^9^ CFU/mL AvX005. After 12 h, the fish were dissected for collection of the liver and kidney. Quantitative real-time polymerase chain reaction (qRT-PCR) was used to analyze the expression of immune genes. Pathological characteristics were observed through H&E staining. The blank control was injected with the same dose of PBS. The concentration of BaX030 was determined by co-cultivation experiments *in vitro*.

Co-cultivation test: AhX040 and BaX030 were cultured overnight. The concentration of AhX040 was adjusted to 1×10^6^ CFU/mL and the concentration of BaX030 was adjusted to 1×10^9^ CFU/g with fermentation medium. Three groups were set up in the experiment: 1×10^9^ CFU/g BaX030 was inoculated into the fermentation medium; 1×10^6^ CFU/mL AhX040 was inoculated into the fermentation medium; 1×10^6^ CFU/mL AhX040 and 1×10^9^ CFU/g BaX030 were simultaneously inoculated into the fermentation medium, and the two bacteria were mixed culture. We took 1 mL of bacterial solution every 4h, spread the plate to count, and drew the growth curve of the co-culture of AhX040 and BaX030. The determination of AvX005 was consistent with the above method.

### Genomic DNA Extraction and Sequencing

Extraction of total bacterial DNA from intestinal samples was used for high-throughput sequencing in grass carp, the samples were tested by a Bioanalyzer system 2100 (Agilent, Palo Alto, USA) and then subjected to sequencing by a PacBio platform and data analysis (Wagner et al., 2016).

### Bioinformatics Analysis

Raw reads were filtered to remove adaptors and low-quality and ambiguous bases, and then paired-end reads were added to tags by the Fast Length Adjustment of Short reads program (FLASH, v1.2.11) (Magoc and Salzberg, 2011) to obtain the tags. The tags were clustered into operational taxonomic units (OTUs) with a cut-off value of 97% using UPARSE software (v7.0.1090) (Edgar, 2013), and chimaera sequences were compared with the Gold database using UCHIME (v4.2.40) (Edgar et al., 2011). Then, OTU representative sequences were taxonomically classified using Ribosomal Database Project (RDP) Classifier v.2.2 with a minimum confidence threshold of 0.6 and trained on the GreenGenes database v201305 by QIIME v1.8.0 (Caporaso et al., 2010). USEARCH_global (Edgar, 2010) was used to compare all tags with the OTUs to obtain the OTU abundance statistics table for each sample. Alpha and beta diversity was estimated by MOTHUR (v1.31.2) (Schloss et al., 2009) and QIIME at the OTU level, respectively. The Venn plots of the OTUs and OTU rank curves were plotted with VennDiagram version 3.1.1. Sample clustering was conducted by QIIME based on UPGMA. LEfSe cluster was conducted by LEfSe. KEGG analysis was performed using PICRUSt software (Wilkinson et al., 2018). Barplots and heat maps of different classification levels were plotted with R package.

### RNA Extraction and qRT-PCR

BaX030 was cultured in the two media for 20 h and centrifuged at 10000 rpm for 5 min to collect approximately 0.1 g of bacteria, and the total RNA of BaX030 was extracted by Trizol method (Yi et al., 2018). The RNA concentration was determined by NanoDrop 2000 spectrophotometer (Thermo Scientific, Waltham, USA), and the reverse transcription kit of the PrimeScriptTM RT reagent Kit with gDNA Eraser (TaKaRa, Dalian, China) was used to perform genomic DNA removal and reverse transcription reactions. The relative expression level of each mRNA was normalized to 16S rRNA as the internal reference, The transcription levels of seven genes, *leuA*, *zwf*, *leuD*, *secG*, *pdk2*, *pck*A and *gerPC*, were detected.

To evaluate the mRNA expression level of fish liver and kidney, we selected the housekeeping gene *β-actin* as the reference gene (Wang et al., 2015; Yang et al., 2016) and selected 5 immune-related genes, immunoglobulin M (*IgM*), complement C3 (*C3*), lysozyme (*LSZ*), interleukin-1β (*IL-1β*), and interleukin 8 (*IL8*)(**Supplementary Table S1**). All primers were synthesized by Sangon Biotech (Shanghai, China) and Primer Premier version 5 (Premier Biosoft International, Palo Alto, USA) was used for primer design.

#### qRT-PCR

The reaction was carried out on an ABI 7500 real-time PCR system (Applied Biosystems, Waltham, USA). The total reaction system was 20 µL in eight tubes: 10 µL of SYBRTM Green Mix (Applied Biosystems, Waltham, USA), 7.8 µL of ultrapure water, 0.6 µL of F primer, 0.6 µL of R primer, and 1 µL of cDNA. The reaction programme was 50°C for 2 min; predenaturation at 95°C for 10 min; and then a total of 40 cycles of 95°C of denaturation for 30 s and 60°C for 1 min for annealing. Finally, the 2^-ΔΔCt^ relative quantitative method was used to compare and analyse the expression levels of gene mRNA in the samples. Each sample was repeated 4 times.

### Protection Experiment of BaX030

A total of 225 healthy grass carp were divided into the feeding group (BA+Ah, BA+Av) and the control group (WT+Ah, WT+Av, WT+PBS). The water temperature was controlled at 25°C, and the volume of each fish tank was 20 L. The feeding group was cultured with the BaX030 strain with fermentation medium, mixed into the feed at 1×10^9^ CFU/g and fed twice a day. After thirty days, 45 fish from each group were injected with the same concentration of AhX040 or AvX005 and observed for seven days. Their cumulative mortality was calculated. The protective effect of the BaX030 strain on grass carp was evaluated according to the relative percentage survival rate (RPS=(1-feeding group mortality rate/positive control mortality rate)×100%). The same amount of PBS was injected as a blank control group.

### Cytotoxicity Test of Macrolactin A on Grass Carp Liver Cells

We added 100 µL (1×10^4^ cells/mL) of L8824 liver cells to the wells of a 96-well plate and cultured them in a cell incubator held consistently at 30°C for 12 h. 10 µL of 20 µM macrolactin A was added to the wells of the plate. The fermentation supernatants of AhX040 and AvX005 were used as controls. The cells were cultured for 48 h, and the cell morphology was observed under an inverted microscope (DMIL, Mannheim, Germany) at 20-times magnification.

## Results

### Growth Curve Determination and Phase Contrast Microscope Observation

**Figure 1:**
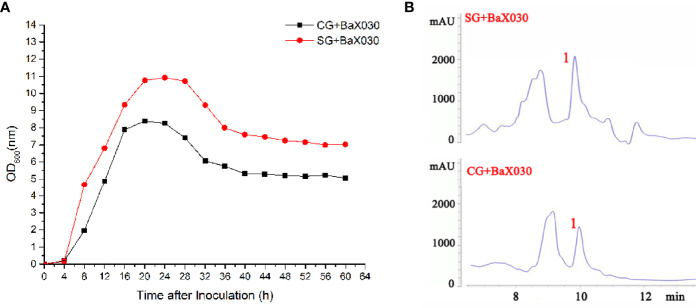
Growth parameters and separation and purification of BaX030. **(A)** The growth curve of CG+BaX030 and SG+BaX030. Datas were mean ± SEM for n=3 biologically independent experiments; **(B)** HPLC chromatograms of BaX030 in different media.

The addition of sucrose promoted cell growth (**Figure 1A**). CG+BaX030 and SG+BaX030 grew slowly in the lag phase (0-4 h); the bacteria grew rapidly in the log phase (4-16 h). After 16 h, the amount of bacteria remained stable, then began to decline after approximately 32 h of incubation. CG+BaX030 began to produce spores at 20 h, Sporulation was delayed by 12 hours in the experimental group (**Supplementary Figure S1**). The addition of sucrose also promoted the biosynthesis of secondary metabolites, and the peak area corresponding to polyketide macrolactin A was increased (**Figure 1B**).

### Yield of Macrolactin A and pH Changes

**Figure 2:**
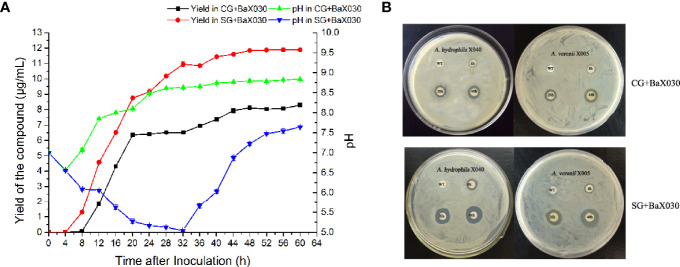
The yield change graph and antibacterial of macrolactin A. **(A)** The yield curve of macrolactin A in two media. Datas were mean ± SEM for n=3 biologically independent experiments; **(B)** The antibacterial experiment of fermentation broth on AhX040 and AvX005 at different time points WT: Original medium without inoculation.

According to the yield curve, the production of macrolactin A had the same obvious upward trend in the early stage, especially in the logarithmic phase (**Figure 2A**). The yield of SG+BaX030 was significantly increased from 8.12 µg/mL to 11.84 µg/mL compared with CG at 48 h. During cultivation, the pH of the experimental group first dropped to 5.02 and then increased, while the control group was basically in an upward trend. The pH level had an observable antibacterial effect on AhX040 and AvX005 at 20 h (**Figure 2B**).

### Statistical Analysis of Differential Proteins

**Figure 3:**
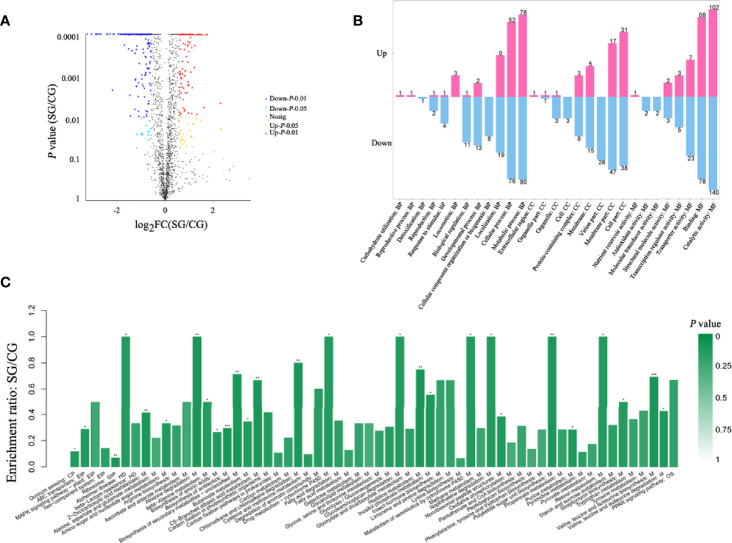
Differential proteomic analysis which SG+BaX030 compared to CG+BaX030. **(A)** The volcano map of differential proteins; **(B)** GO analysis of the differential proteins; **(C)** KEGG analysis, biological pathways involved in differential proteins. ***P < 0.001; **P < 0.01; *P < 0.05.

Whole protein was extracted from BaX030 in the LB medium and the fermentation medium, and SDS-PAGE showed clear bands (**Supplementary Figure S2**). The total number of proteins was identified as 2055 by iTRAQ, including 143 upregulated proteins and 307 downregulated proteins (**Figure 3A**). Gene Ontology (GO) analysis found that compared to CG+BaX030, the proteins of SG+BaX030 were significantly enriched in the molecular function (MF) process of catalytic activity. The second was the biological process (BP) of metabolism (**Figure 3B**).

### Analysis the Differential Proteins by KEGG Pathway

**Figure 4:**
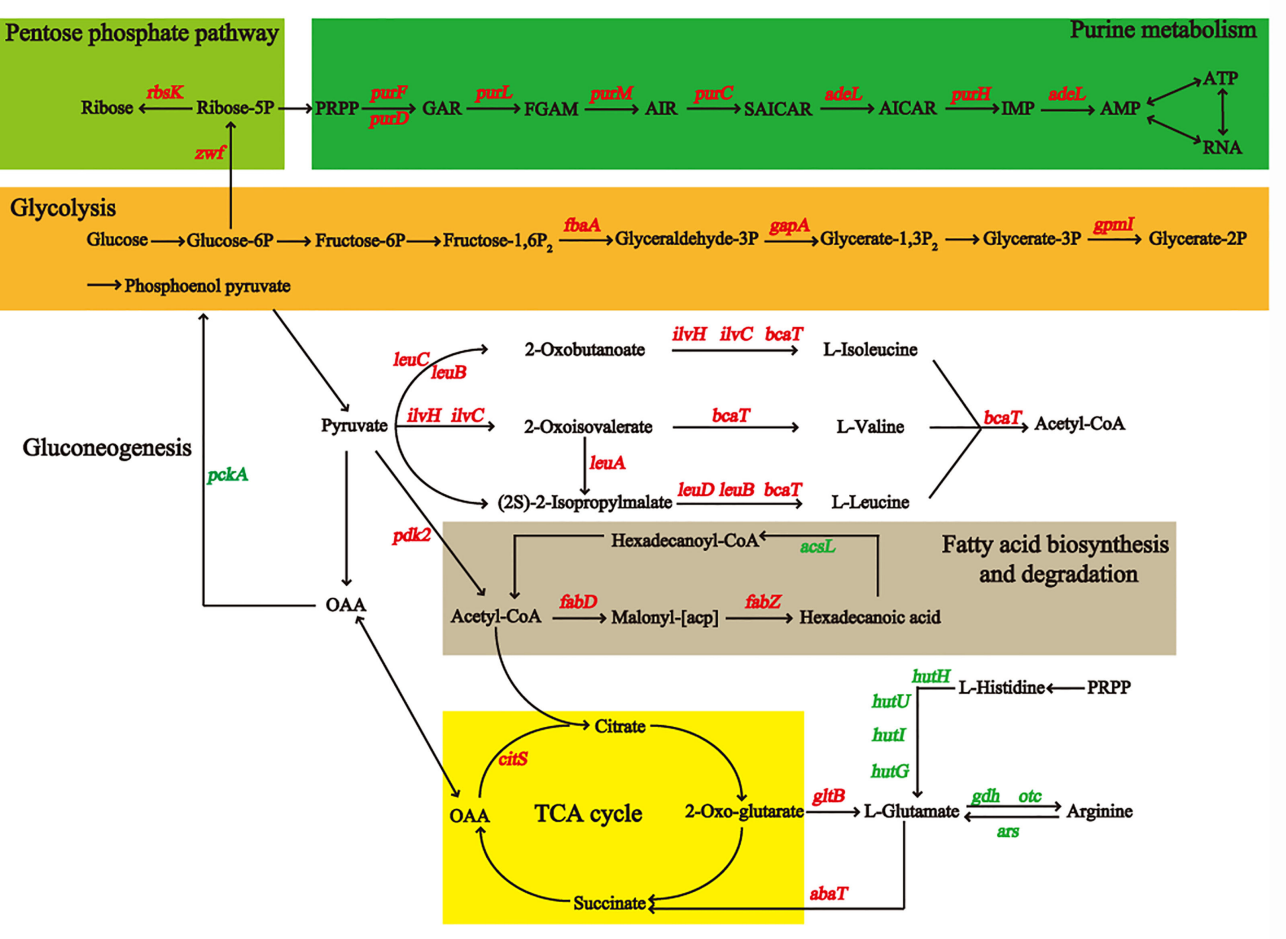
The diagram of metabolic pathways may be affected by addition of sucrose for BaX030, including glycolysis/gluconeogenesis, TCA cycle, pentose phosphate pathway, purine metabolism, fatty acid/amino acid synthesis and metabolism, etc. The marked genes in red corresponding to the proteins were up-regulated, and the green were down-regulated.

The metabolic pathways of 450 differentially expressed proteins were classified by Kyoto Encyclopedia of Genes and Genomes (KEGG). We found that the most different proteins were involved in metabolic processes (M) (**Figure 3C**). We detected the upregulated genes *fbaA*, *gapA*, and *gpmI* in the glycolysis pathway and the downregulated gene *pepck* in the gluconeogenesis pathway (**Figure 4**) (**Supplementary Table S2**), which contributed to the enrichment of pyruvate and acetyl-CoA. They provided an important raw material for the synthesis of fatty acids, and the significant increase in macrolactin A was largely dependent on the accumulation of fatty acids. A large number of proteins were upregulated in the pentose phosphate and the tricarboxylic acid cycle pathway, which supplied cells with sufficient carbon scaffolds and ATP.

### Newly Discovered Potential Functional Proteins That Importantly Impact the Expression of Macrolactin A

**Figure 5:**
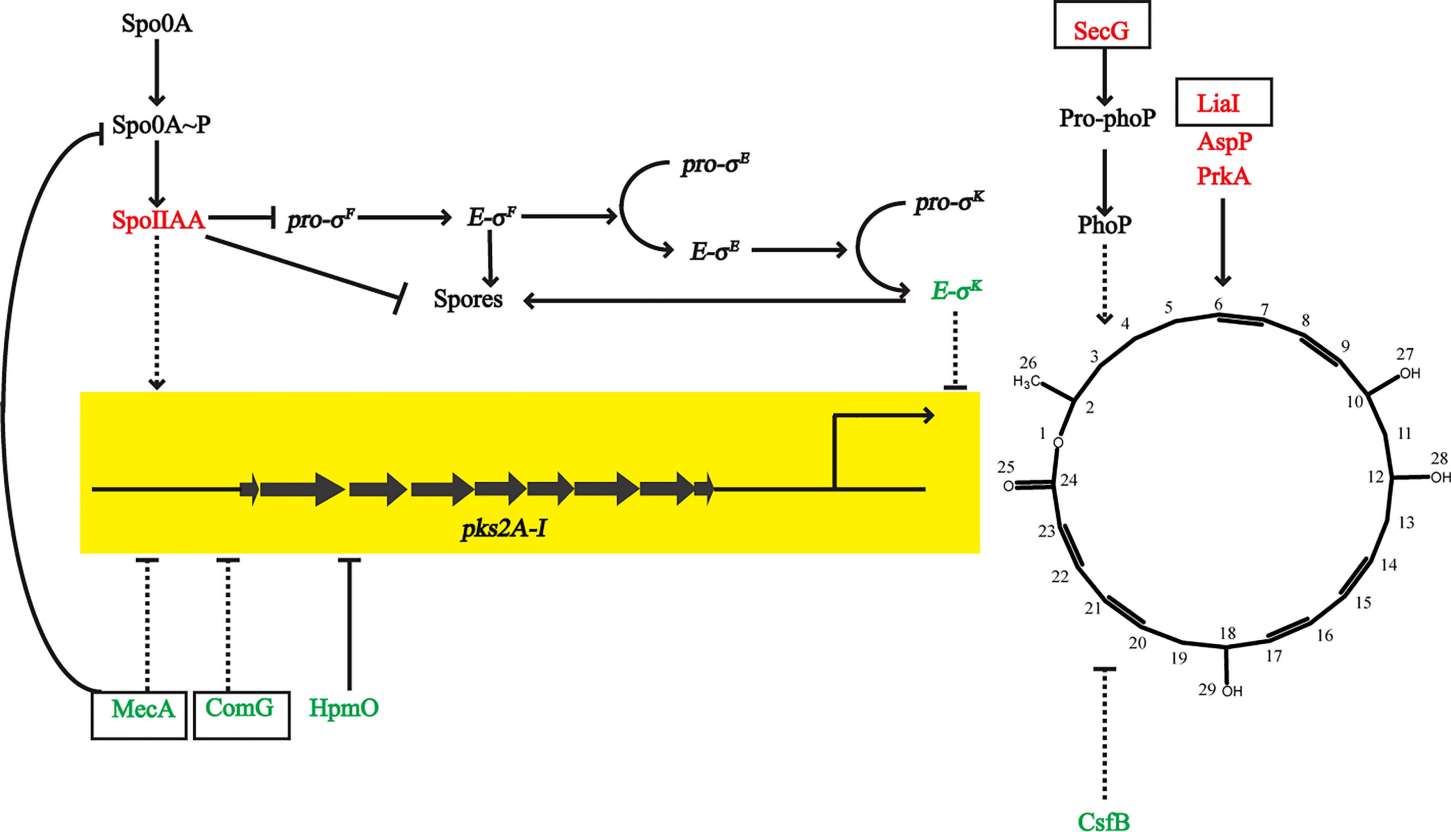
Differential proteins that affected the expression of macrolactin A. Arrows and T-shapes indicated positive and negative regulation, respectively. The solid indicate direct effect, and the dotted line indicate indirect effect. The black box marks represented new discovered and important proteins in this study.

According to proteomic data, the expression of the *pks* operon was increased after adding sucrose (gene cluster 9) (**Figure 5**), so we searched for proteins related to macrolactin A to further evaluate their potential impact, including several newly discovered proteins. The anti-sigma F factor encoded by *spoIIAB* was upregulated, which was involved in the formation of spores in stage II. It inhibited the release of the precursor-sigma F factor (*pro-σ^F^*) and led to the downregulation of RNA polymerase-*σ^E^* (*E-σ^E^*). LiaI is a small membrane protein with two transmembrane helices that may be able to counteract antibiotic-induced damage, which is beneficial to the production of metabolites. AspP showed the same expression trend as macrolactin A, which proved its positive effect on macrolactin A. The preprotein translocase subunit SecG promoted the ATP-dependent translocation of precursor PhoB (Pro-phoB) and then affected macrolactin A.

### qRT-PCR Verification

The three bands of 23 S, 16 S and 5S rRNA were clear (**Supplementary Figure S3**), and the quality of the extracted RNA was good. The qRT-PCR results showed that the transcription fold increases of *leuA*, *zwf*, *leuD*, and *secG* were 3.61, 2.07, 2.96 and 5.06 times, respectively. The transcription fold values of *pdk2*, *pckA* and *gerPC* were 0.48, 0.43 and 0.31 times, respectively, which was also consistent with the same trend observed in the proteomics data (**Supplementary Figure S4**; **Supplementary Table S2**).

### Analysis of the Structural Characteristics of the Intestinal Microbiome

**Figure 6:**
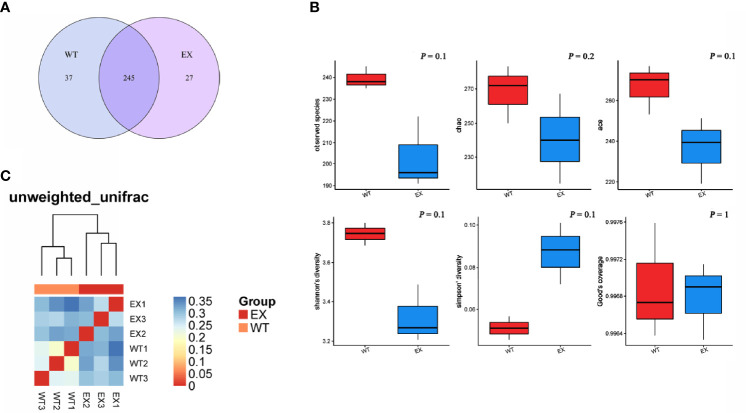
The structural characteristics of the intestinal microbiome. **(A)** Venn diagram of two groups intestinal samples of BaX030-added or not based on OTU; **(B)** Diversity analysis of the intestinal microbiota in grass carp between healthy and diseased groups; **(C)** The heat map of Beta diversity.

The Venn diagram showed that the total number of OTUs was 245 in the two groups, and the unique OTU numbers of the EX group and the WT group were 27 and 37, respectively (**Figure 6A**), which indicated that BaX030 can change the composition of the intestinal flora of grass carp. An α diversity analysis showed that the coverage of intestinal samples exceeded 99% in the EX and WT groups. The observed species index, Chao index, Ace index, and Shannon index of the EX group were lower than those of the WT group, and the Simpson index was greater than that of the WT group. The OTU rank curve showed that the experimental group had a faster downward trend than the control group. The results demonstrated that BaX030 can increase the proportion of the dominant flora and reduce the diversity of the community in the gut microbiome (**Figure 6B**, **Supplementary Figure S5**). We found that the experimental group and the control group were clustered on their respective branches by clustering trees, their composition structure was different (**Figure 6C**).

### Analysis of the Abundance of Intestinal Microbiome

**Figure 7:**
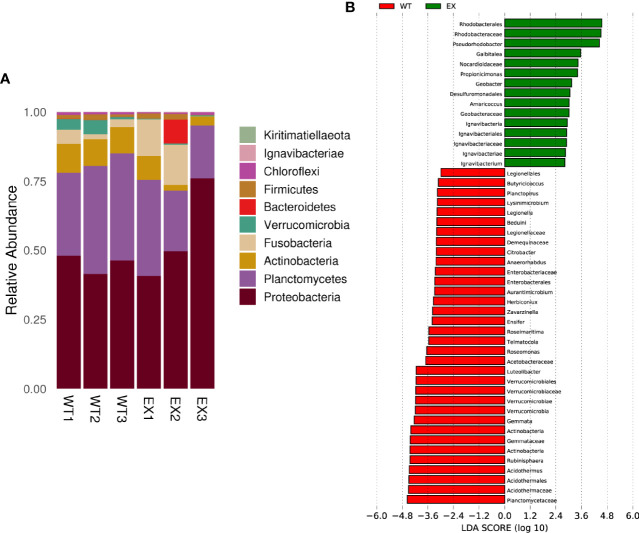
Effects of BaX030 on the intestinal flora of grass carp. **(A)** The composition of bacterial community on total 6 samples at phylum level; **(B)** Effect of BaX030 on the intestinal microbiome of grass carp by LefSe analysis.

A total of 10 bacterial phyla were identified from in 6 samples. *Proteobacteria*, *Planctomycetes*, *Actinobacteria*, *Fusobacteria*, *Verrucomicrobia*, *Bacteroidetes*, *Firmicutes* and *Chloroflexiare* were phyla with an abundance of more than 0.1%. *Proteobacteria* (55.48 ± 18.46%) and *Planctomycetes* (25.32 ± 8.44%) dominated the taxonomic composition in the EX and WT (45.24 ± 3.38% and 36.04 ± 5.09%, respectively) groups (**Figure 7A**). The control group showed a relatively high average abundance of *Actinobacteria* (9.80% ± 0.49%), whereas the treatment group showed a relative dominance of *Fusobacteria* (9.27 ± 7.96%). Additionally, the pathogens *Aeromonas* were reduced from 5.2% ± 1.32% to 3.37 ± 1.19%.

LEfSe was performed to characterize the microbial communities exhibiting significant differences in abundance between the WT and EX groups. The results showed that the relative abundances of the families *Rhodobacteraceae*, *Nocardioidaceae*, *Geobacteraceae* and *Ignavibacteriaceae* were significantly higher in the EX group, while the relative abundances of the families *Planctomycetaceae*, *Actinomycetaceae*, *Verrucomicrobiaceae*, and *Enterobacteriaceae* were significantly increased in the WT group (*P* < 0.05) (**Figure 7B**), which suggested that metabolic changes occurred in the intestinal microbiota of grass carp caused by the feeding of BaX030.

### Influence of BaX030 on Specific Immunity in Grass Carp

**Figure 8:**
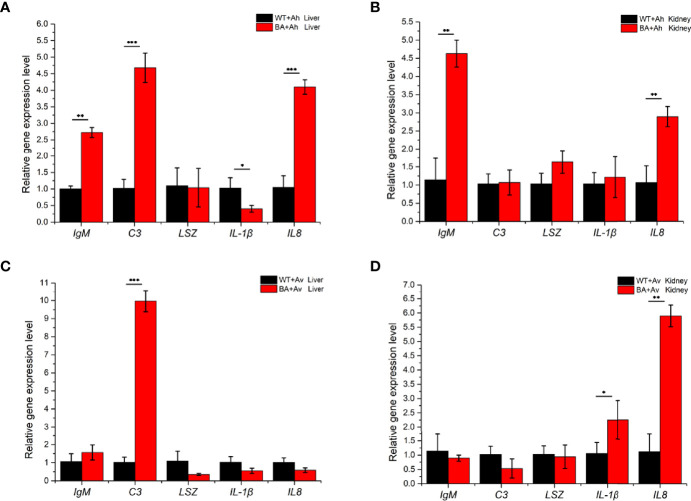
Analysis of immune-related gene expression in grass carp that fed with BaX030 after infection with AhX040 and AvX005. **(A, B)** Expression of immune-related genes in the liver and kidney after infection with AhX040; **(C, D)** The gene expression levels of immune-related cytokines in liver and kidney at post-infection AvX005. WT+Ah: grass carp+AhX040; BA+Ah: grass carp*+*BaX030+AhX040; WT+Av: grass carp+AvX005; BA+Av: grass carp*+*BaX030+AvX005. Datas were mean ± SEM for n=4 biologically independent experiments. Statistical analysis were performed using one-way ANOVA. ***P <0.001; **P <0.01; *P <0.05.

The expression of immune-related genes in the liver and kidney was determined by qRT-PCR (**Supplementary Figure S6**). In the liver, the gene expression levels of *IgM* and *IL8* in the BA+Ah group were significantly higher than those in the BA+Av and control groups, and the expression levels of *C3* were significantly improved in the two groups (**Figure 8**). In the kidney, the expression level of *IgM* was higher in the BA+Ah group than in the other groups. However, the upregulation of the *IL8* gene was lower than that in the BA+Av group. The expression level of *IL-1β* was increased only in the BA+Av group.

### Pathological Observation of Grass Carp

The WT+Ah group began to show signs of stress at 12 h, including obvious congestion of the abdomen and pelvic fins, red and swollen anus, and scale loss. The WT+Av group mainly manifested in slight congestion of the abdomen and pelvic fins and red and swollen anus at 48 h. However, the symptoms were relieved in the experimental group (**Supplementary Figure S7**).

**Figure 9:**
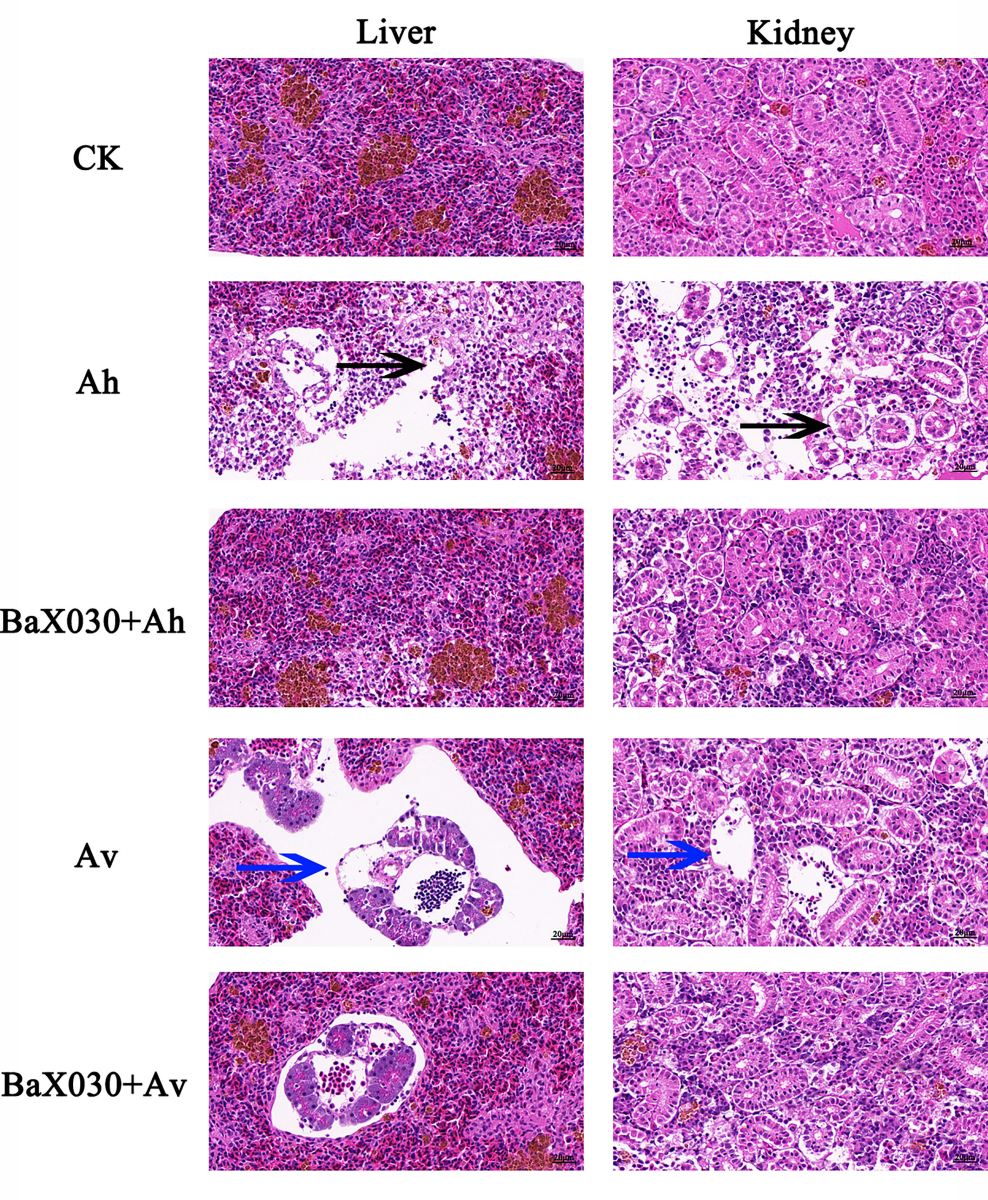
The pathological changes of the liver and kidney in grass carp were observed by H&E staining (400×). The black arrow indicated the hepatocytes were exfoliated by apoptosis, the blue arrow indicated the nephrocyte were exfoliated to form large cavities.

H&E staining results showed that the hepatocytes of grass carp infected with AhX040 were exfoliated by apoptosis and separated from the outer membrane of blood vessels (**Figure 9**); the nephrocytes were severely vacuolated, denatured and necroti, the intercellular space was enlarged. The vascular cells infected with AvX005 showed vacuolar degeneration and separation from surrounding tissues. A large number of cells were exfoliated to form large cavitie. The corresponding pathological signs were improved in the experimental group. The results showed that BaX030 could protect the immune organs and reduce the virulence of the pathogen.

### Protection Experiment of BaX030

The fermentation supernatant of the SG+BaX030 group was not toxic to liver cells, as indicated in cell experiments. The cells were contracted and round, which proved that they all had died in the Ah group and the Av group. In the macrolactin A group, the cell arrangement was loose, and the number of cells was increased compared to the control, which had a certain protective effect on cells (**Supplementary Figure S8**).

**Figure 10:**
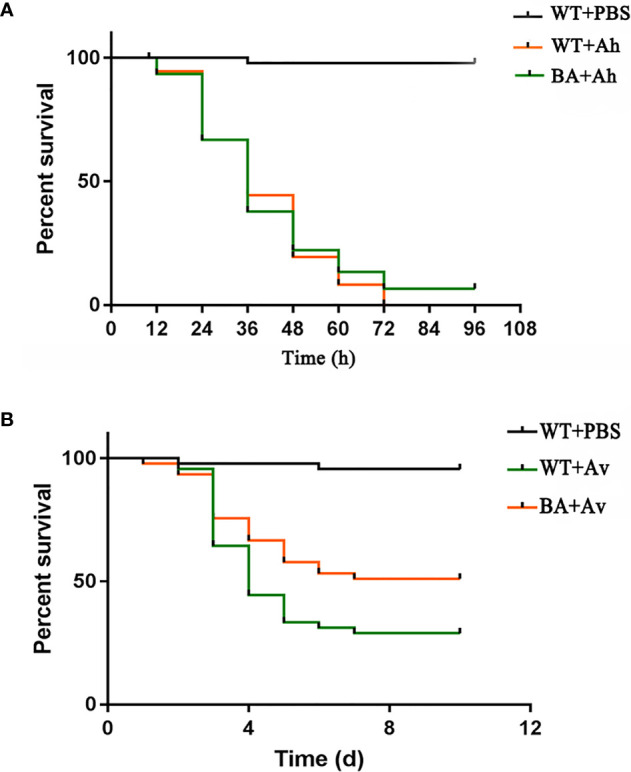
The cumulative survival rate of grass carp in control group and experimental group. **(A)** The cumulative survival rate of grass carp after being infected with AhX040; **(B)** The cumulative survival rate of grass carp after being infected with AvX005.

When the initial dose of 1×10^9^ CFU/g BaX030 was co-cultured with 1×10^6^ CFU/mL AhX040 or 1×10^9^ CFU/mL AvX005, BaX030 could completely inhibit the growth of two pathogens (**Supplementary Figure S9**). After intraperitoneal injection of AhX040 and AvX005, grass carp were continuously observed for 4 and 9 days, respectively, and the cumulative survival rate was determined. The results showed that the cumulative survival rate of the BA+Ah group was 20.0%, which was 13.3% lower than that of the control group, and the RPS value was 14.3%. The cumulative survival rate of the BA+Av group was 51.1%, which was 22.2% lower than that of the control group, and the RPS value was 37.1% (**Figure 10**). Therefore, BaX030 can reduce the damage caused by two pathogens in grass carp.

## Discussion

Many antibiotics had stimulating activity at low concentrations, which is called “hormesis”. They induced transcriptional activation and promoted the growth and biofilm formation of *Bacillus* (Hoffman et al., 2005; Davies et al., 2006). Sucrose stimulated bacterial growth, which led to an increase in the production of macrolactin A (**Figures 1A**, **2A**). Macrolactin A may regulate its own “hormesis” effect by inducing bacteria to produce certain signalling molecules when irritated by sucrose (Liu et al., 2018). The total production of bacteria and macrolactin A was higher than that of the control group. We tried to add other carbon sources, including glucose, fructose, maltose, lactose, and starch, but the effect was not as good as that of sucrose. BaX030 was the low utilization of some carbon sources due to different bacterial growth characteristics, such as maltose, lactose, etc. Another possible reason was that although other carbon sources were added to promote the growth of the strain, it did not lead to the increase in the yield of the target product macrolactin A. Sucrose additional was an effective strategy because it is a cheaper carbon source and readly available in nature. *B. amyloliquefaciens* has higher sucrose hydrolysis activity, a lower free sugar consumption rate, and can use sucrose to grow rapidly, resulting in a higher biomass yield (Bhatia et al., 2018). In addition, polyketides often need to carry other functional groups to have strong biological activity on the carbon atom framework, such as hydroxyl groups, carboxyl groups, double bonds, amino acid methylation, or glycoside formation. Macrolactin A constitutes a class of 24-membered macrolide compounds, and it had a good antibacterial effect because its structure contained a large number of carbon-carbon double bonds and hydroxyl groups (Ortiz and Sansinenea, 2020).

*A. hydrophila* and *A. veronii* can adhere to the intestinal tract and at the same time produce toxins to make fish sick (Wang et al., 2016; Zhang et al., 2019; Zhang et al., 2020). Our results showed that pathological damage mainly included damage to the liver, kidney and other immune organs, and the expression of related immune genes was downregulated, which ultimately reduced the resistance of grass carp to pathogen invasion (**Figures 8**, **9**). *B. amyloliquefaciens* can regulate fish growth and immunity by colonizing fish intestines, where it can compete with pathogenic microorganisms for adhesion sites and inhibit the proliferation of *Aeromonas* species because of its high rate of adhesion to the intestinal mucosa (Thankappan et al., 2015; Santos et al., 2021). Many studies have proven that *Bacillus* can produce antibacterial active substances and reduce mortality in aquaculture (Pinchuk et al., 2001; Nayak, 2010). Therefore, *Bacillus* can colonize in fish intestines and can also produce macrolactin A. To maximize the probiotic function of BaX030, we considered increasing the amount of the strain applied or cotreating fish with other probiotics as a means of improving the resistance of the fish to pathogenic bacteria.

The dominant phyla contained *Proteobacteria*, *Firmicutes*, *Bacteroidetes* and *Actinobacteria* in the experimental group and the control group. As the core microbiota of the intestine in grass carp, these bacteria can effectively regulate the structure of the intestinal microbiome and affect its healthy growth (Han et al., 2010; Roeselers et al., 2011; Yang et al., 2019). These bacteria contain a diverse functional repertoire, including chemoorganotrophic ability to utilize environment-derived fatty acids, aromatics, carbohydrates, and peptides (Zhou et al., 2020). Because of these abilities *Proteobacteria* in high abundance indirectly promoted the adaptability of grass carp to the environment. Moreover, *Proteobacteria* and *Actinobacteria* also had a certain positive effect on the production of polyketides, including macrolactin A (Desriac et al., 2013; Buijs et al., 2019). Li et al. (2013) found that the relative abundance of *Firmicutes* and *Bacteroides* affects the growth of fish; the ratio was higher, and the growth rate was faster. The ratio was decreased after the grass carp were fed BaX030, but the two groups were not different in terms of body weight. Therefore, the validity of this potential marker is worth discussing. Currently, it is difficult to relate the ratio of *Firmicutes*/*Bacteroidetes* to the growth status in grass carp (Magne et al., 2020). In addition, the abundance of probiotic *Gemmobacter* in the experimental group (16.9%) was higher than that in the control group (10.4%), and the abundance of potential pathogens, such as *Aeromonas*, was lower than that in the control group, which indicated that the intestinal microbiome of grass carp fed BaX030 had a better structure and a healthier composition.

The interaction between probiotic *bacilli* and fish immune cells can lead to an enhanced immune response (Sangma and Kamilya, 2015; Gobi et al., 2018). Previous reports have proven that adding *B. amyloliquefaciens* to feed can enhance the immunity and disease resistance of *Nile tilapia*, *Labeo rohita* and zebrafish against *A. hydrophila* infection (Nandi et al., 2018; Lin et al., 2019; Kuebutornye et al., 2020). *IL-1β* and *IL8* are the two main proinflammatory cytokines in fish, and they can balance the inflammatory response to bacterial infection (Reis et al., 2012; Wang et al., 2019). The biological activity of *IL8* can attract and activate neutrophils. After contacting *IL8*, neutrophils will undergo morphological changes, move towards the reaction site, and release a series of active products (Wang et al., 2017). *C3* had the highest degree of upregulation in the liver, which proved that *B. amyloliquefaciens* can potentially improve liver stress tolerance (Singh et al., 2017). The *C3* and *IL8* levels of the liver and kidney of grass carp were significantly increased in the experimental group, indicating that the upregulation of two immune factors was essential for the inhibition of AhX040 and AvX005. *LSZ* is a small-molecule alkaline enzyme that hydrolyses mucopolysaccharides and can block the invasion of foreign pathogenic bacteria (Derde et al., 2017). *IgM* is the immunoglobulin with the largest molecular weight and can lyse bacteria and neutralize viruses. Its deficiency lead to a tendency for sepsis and/or pulmonary oedema caused by gram-negative bacteria to develop (Bilal et al., 2016; Su et al., 2018). This finding was consistent with our results; BaX030 was beneficial to the improvement of immune factor *IgM* and reduced the pathological sign in grass carp.

When the *prkA* gene was destroyed, the activity of *B. amyloliquefaciens* was reduced (Liu et al., 2013), so the significantly upregulation of PrkA may have promoted the production of secondary metabolites. The ComGA-G operon of the binding and transport of DNA (Briley et al., 2011) in *Bacillus* and the two-component stimulating factor CsfB (Lu et al., 2019) with quorum sensing activity showed the opposite expression trend of macrolactin A, which suggested their negative effects on its production. The expression of HPMO can degrade hexadecane and cause fatty acids to not be converted into polyketides efficiently, which was not conducive to the expression of the *pks* operon (Yu et al., 2013). The adapter protein MecA targeted the transcription factor ComK and was then degraded by the ClpC/ClpP proteolytic complex to negatively regulate the activities of *Bacillus*, including directly binding and inhibiting the transcriptional activity of Spo0A-P, as well as inhibiting the biofilm formation activity (Prepiak et al., 2011). It was worth mentioning that we also detected the corresponding up-regulation of NRPS in the proteomics data, especially involving the synthesis of fengycins (**Supplementary Table S2**). However, it may not have a direct antibacterial effect through using an agar diffusion test, so we probably removed it in the separation and purification. Instead, it competed with certain sites in the quorum sensing system for *A.hydrophila* and *A.veronii* decolonization (Piewngam et al., 2018). The mechanism was interesting and can be studied further.

In conclusion, the study revealed that sucrose improved the growth of BaX030, affected key functional proteins and potential regulators of macrolactin A, and enhanced the antibacterial activity. It provided a detailed database of key primary metabolism, secondary metabolism and regulatory proteins related to biosynthesis. The yield of target products can be further improved by optimizing the culture medium and genetic modification in the industry. Protection experiments showed that BaX030 was not only beneficial to the intestinal flora in grass carp, but also modulated the immune responses and function against AhX040 and AvX005 infections. The probiotic *Bacillus* has potential application value as a feed additive and good prospects for its application in aquaculture, immunity, and control of fish diseases. Our study laid a foundation for establishing the relationship between economic traits and intestinal microbiota.

## Data Availability Statement

The datasets presented in this study can be found in online repositories. The names of the repository/repositories and accession number(s) can be found in the article/**Supplementary Material**.

## Ethics Statement

This study had been reviewed and approved by the Ethics Committee of Hunan Normal University, the approval number was 2021388.

## Author Contributions

PZ wrote the manuscript. WC and ZZ conducted the experiments. KZ and SL participated in sample processing. SH and LX analyzed data. XD conceived the research and completed the revision of the manuscript. All authors read and approved the manuscript.

## Funding

This work was supported by the National key Research and Development program of China (2017YFD0201201), the National Natural Science Foundation of China (31370116).

## Conflict of Interest

The authors declare that the research was conducted in the absence of any commercial or financial relationships that could be construed as a potential conflict of interest.

## Publisher’s Note

All claims expressed in this article are solely those of the authors and do not necessarily represent those of their affiliated organizations, or those of the publisher, the editors and the reviewers. Any product that may be evaluated in this article, or claim that may be made by its manufacturer, is not guaranteed or endorsed by the publisher.
