# Supplementary material for: Comparative Study of Bacillus amyloliquefaciens X030 on the Intestinal Flora and Antibacterial Activity Against Aeromonas of Grass Carp
Source: Front Cell Infect Microbiol. 2022 Jan 25;12:815436. doi: 10.3389/fcimb.2022.815436 (PMC8821659; doi:10.3389/fcimb.2022.815436)
Supplement: Supplementary file 1 [file DataSheet_1.pdf]

Supplementary Material

Comparative study of *Bacillus amyloliquefaciens* X030 on the intestinal flora and antibacterial activity against *Aeromonas* of grass carp

Pengji Zhou, Wenhui Chen, Zirong Zhu, Kexuan Zhou, Sisi Luo, Shengbiao Hu, Liqiu Xia, Xuezhi Ding*

State Key Laboratory of Developmental Biology of Freshwater Fish, Hunan Provincial Key Laboratory of Microbial Molecular Biology, College of Life Science, Hunan Normal University. Changsha 410081, China.

Pengji Zhou 1192131669@qq.com

Wenhui Chen 1604618605@qq.com

Zirong Zhu 1832499112@qq.com

Kexuan Zhou 916172868@qq.com

Sisi Luo 2517072664@qq.com

Shengbiao Hu 6498034@qq.com

Liqiu Xia xialiqiu@hunnu.edu.cn

Xuezhi Ding* dingxuezhi@hunnu.edu.cn

*** Correspondence to:**

Xuezhi Ding

State Key Laboratory of Developmental Biology of Freshwater Fish, Hunan Provincial Key Laboratory of Microbial Molecular Biology, College of Life Science, Hunan Normal University. Changsha 410081, China.

E-mail: dingxuezhi@hunnu.edu.cn

Supplementary Figure List

Fig. S1 The strain morphology at 20 h by phase contrast microscope (100×)

Fig. S2 The whole protein extraction and SDS-PAGE analysis of BaX030 at 20h. M: protein marker; Lane 1-2: CG+BaX030; Lane 3-5: SG+BaX030

Fig. S3 Total RNA extraction of BaX030. Lane 1: CG+BaX030; Lane 2: SG+BaX030

Fig. S4 qRT-PCR analysis of changes of key genes in the transcription level. Datas were mean ± SEM for n=4 biologically independent experiments. Statistical analysis were performed using one-way ANOVA. ***$P < 0.001$; **$P < 0.01$; *$P < 0.05$

Fig. S5 OTU rank curve of intestinal microflora abundance in grass carp

Fig. S6 RNA extraction from the liver and kidney of grass carp that infected with AhX040 and AvX005, respectively. Lane 1: WT+Ah Liver; Lane 2: WT+Ah Kidney; Lane 3: BA+Ah Liver; Lane 4: BA+Ah Kidney; Lane 5: WT+Av Liver; Lane 6: WT+Av Kidney; Lane 7: BA+Av Liver; Lane 8: BA+Av Kidney

Fig. S7 Protective test of BaX030 on grass carp and the symptoms of infection with AhX040 and AvX005

Fig. S8 Effects of macrolactin A on hepatocytes L8824 in grass carp

Fig. S9 Growth curves of the initial inoculum concentration of 1×10^9 CFU/g BaX030 and 1×10^6 CFU/mL AhX040/1×10^9 CFU/mL AvX005 were obtained by single culture and co-culture

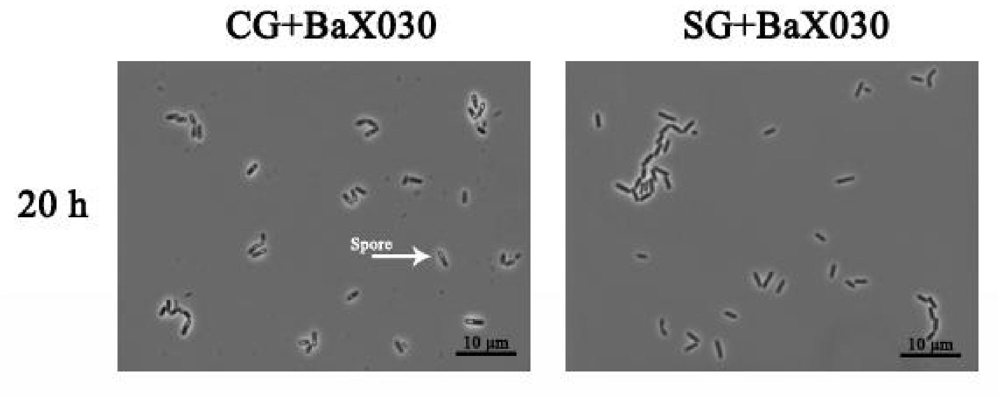

Fig. S1 The strain morphology at 20 h by phase contrast microscope (100×)

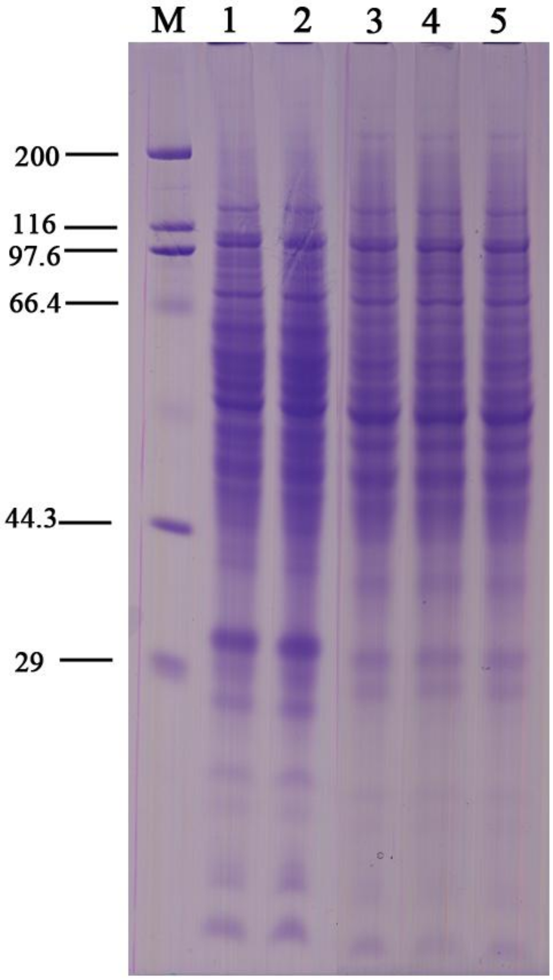

Fig. S2 The whole protein extraction and SDS-PAGE analysis of BaX030 at 20h. M: protein marker; Lane 1-2: CG+BaX030; Lane 3-5: SG+BaX030

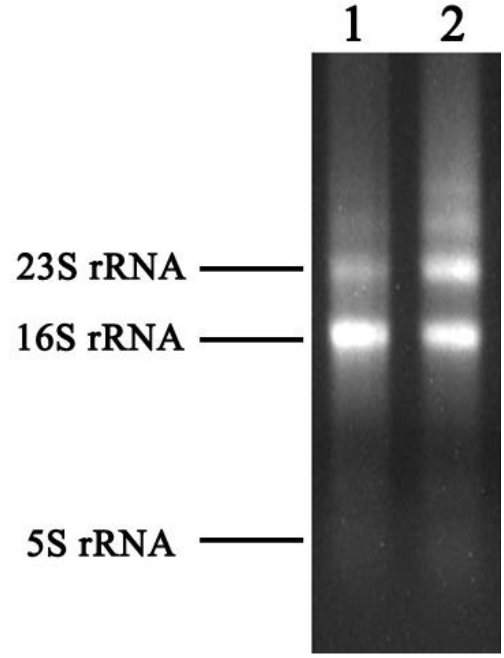

Fig. S3 Total RNA extraction of BaX030. Lane 1: CG+BaX030; Lane 2: SG+BaX030

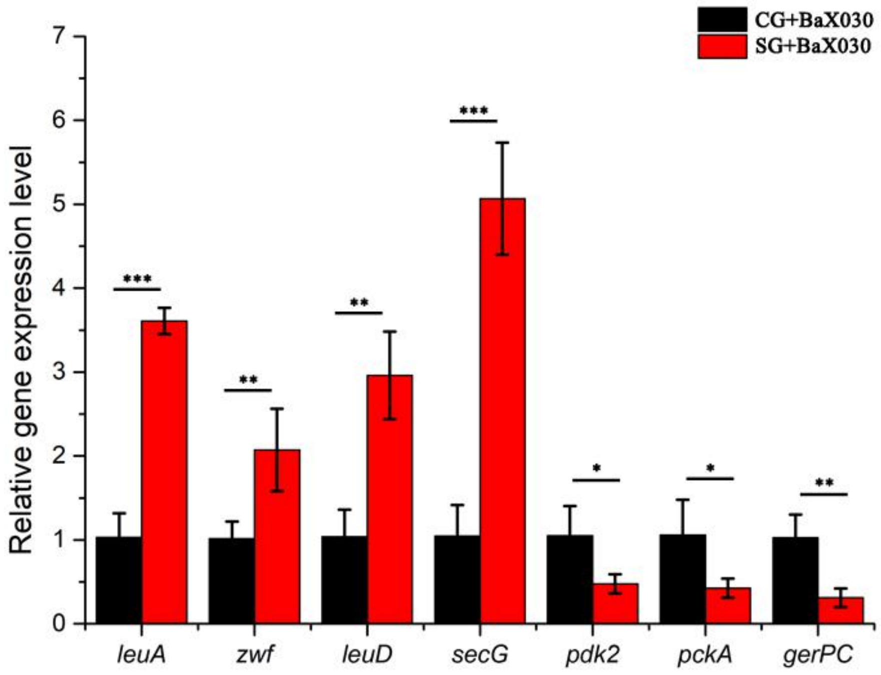

Fig. S4 qRT-PCR analysis of changes of key genes in the transcription level. Datas were mean ± SEM for n=4 biologically independent experiments. Statistical analysis were performed using one-way ANOVA. ***$P < 0.001$; **$P < 0.01$; *$P < 0.05$

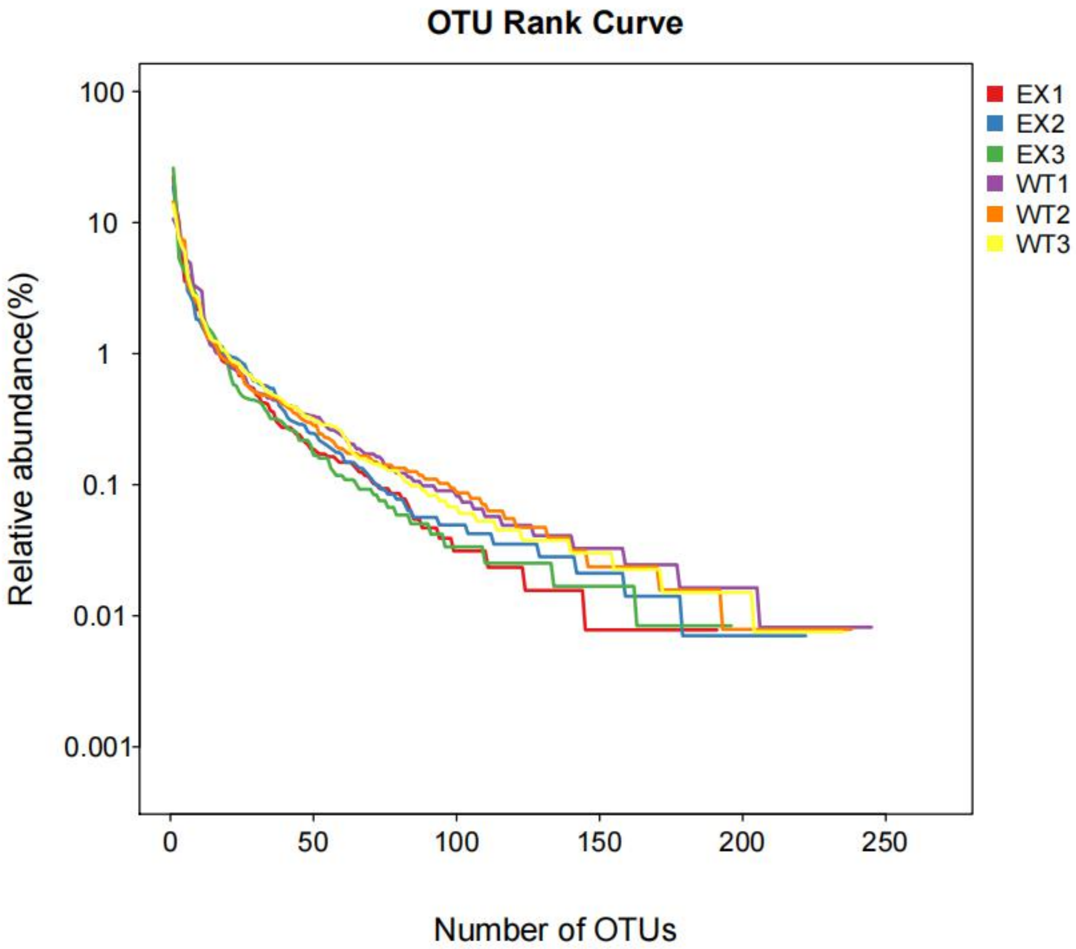

Fig. S5 OTU rank curve of intestinal microflora abundance in grass carp

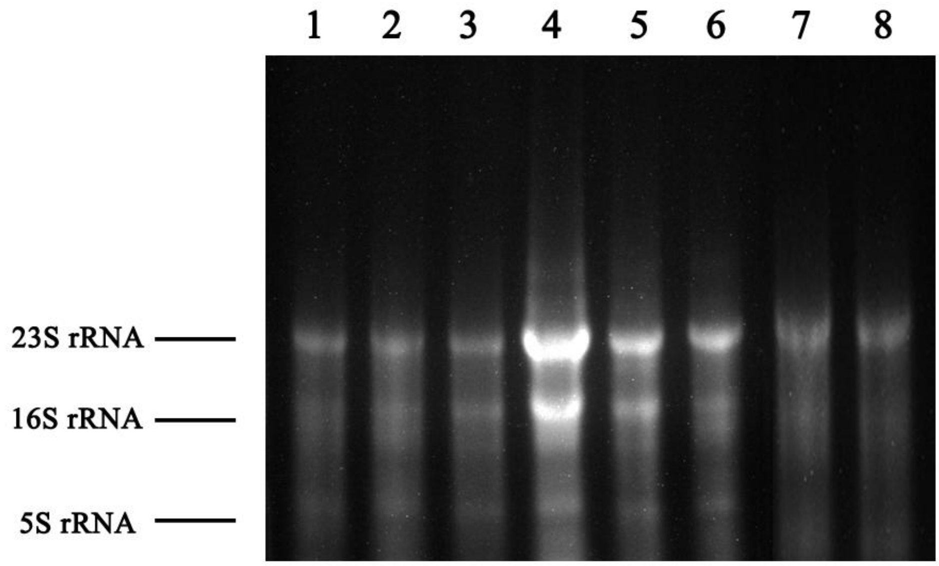

Fig. S6 RNA extraction from the liver and kidney of grass carp that infected with AhX040 and AvX005, respectively. Lane 1: WT+Ah Liver; Lane 2: WT+Ah Kidney; Lane 3: BA+Ah Liver; Lane 4: BA+Ah Kidney; Lane 5: WT+Av Liver; Lane 6: WT+Av Kidney; Lane 7: BA+Av Liver; Lane 8: BA+Av Kidney

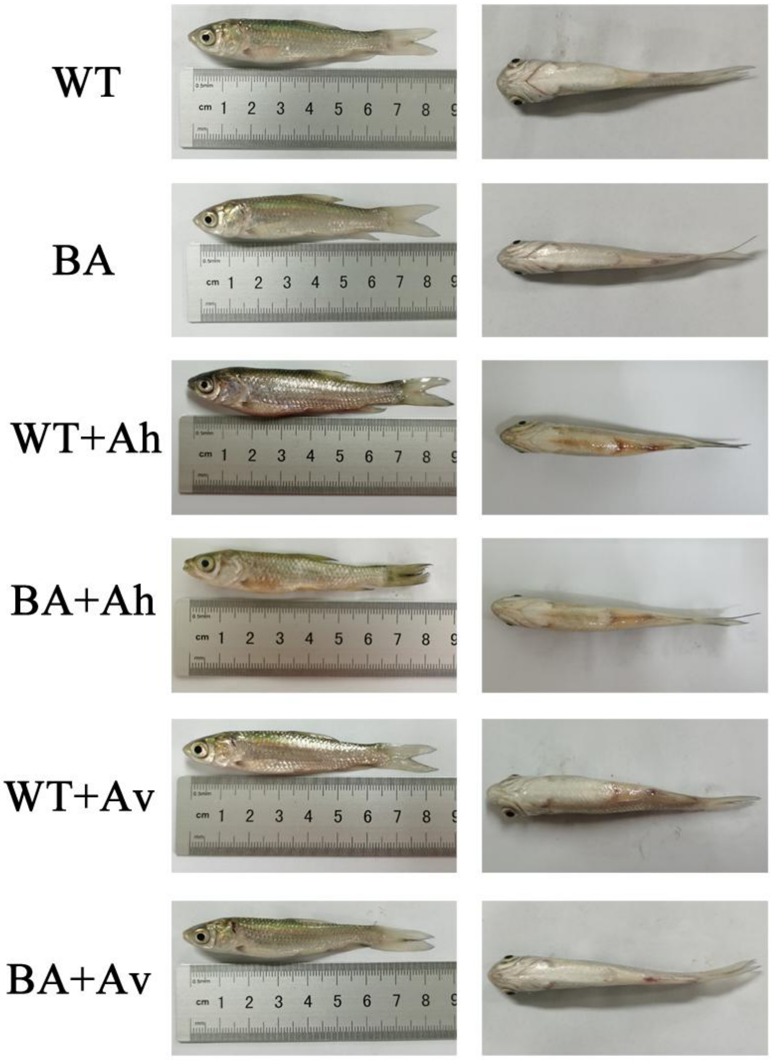

Fig. S7 Protective test of BaX030 on grass carp and the symptoms of infection with AhX040 and AvX005

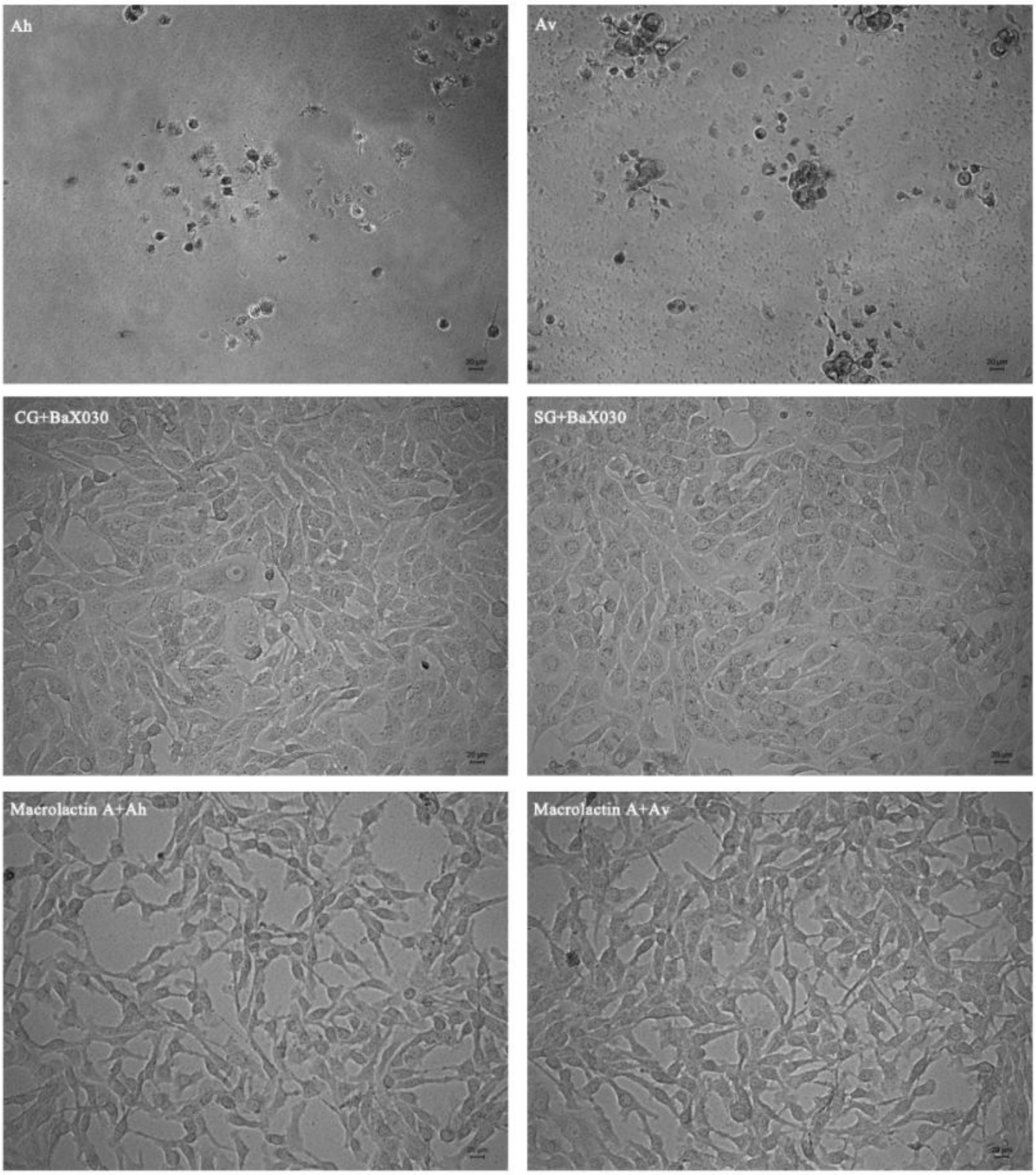

Fig. S8 Effects of macrolactin A on hepatocytes L8824 in grass carp

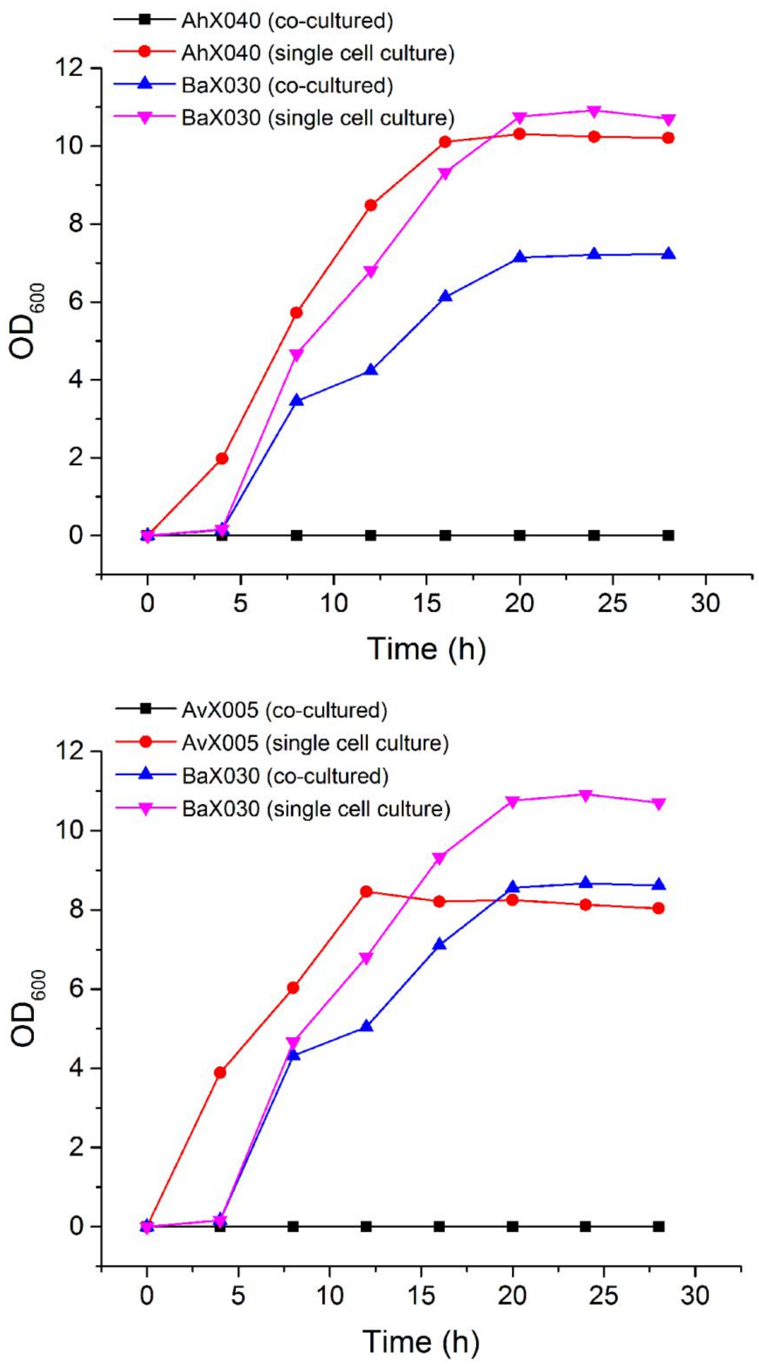

Fig. S9 Growth curves of the initial inoculum concentration of 1×10^9 CFU/g BaX030 and 1×10^6 CFU/mL AhX040/1×10^9 CFU/mL AvX005 were obtained by single culture and co-culture

Supplementary Table List

Table S1 qRT-PCR primers in this study

Table S2 The different protein abundances in the two media

Table S1 qRT-PCR primers in this study

Primers	Sequences (5' to 3')
16S rRNA	F:CAACGCGAAGAACCTTACCA<br>R:CACGAGCTGACGACAACCA
leuA	F:TCCGAGATCATCGTGGCA<br>R:GCGAGCGTCATCAACCTG
zwf	F:GACTGTCTGCTCGGTGAT<br>R:TAATTCGGAGAAAGGGTT
leuD	F:GCAAATGACGGAGCAATG<br>R:GGTGAGCCTGACCCTGAA
secG	F:AACGGTTGTGCGGTGAAG<br>R:TTCAAACCAGCAAAAGCG
pdk2	F:ATGTTCTGGCTGAAGTGC<br>R:AGGTGCGTCAAACGTAAT
pckA	F:CATTGAAGGCGGATGTTA<br>R:AATCGGATATGCGGCTCT
gerPC	F:CGTTCCTTCCAGCCGTTCA<br>R:ATCAATCCGTTTCATCCTA
β-acting	F: GCTATGTGGCTCTTGACTTCG<br>R: GGGCACCTGAACCTCTCATT
IgM	F: TGGTCATCAGGTGGCAAATAC<br>R: GCGGCTGTCTTCCATTCTT
C3	F: AATACGCCATTCCTGAGGTTTC<br>R: GCTTCAATGCCAACTGTCAGAC
LSZ	F: TTCGACAGCAAAACAGGACA<br>R: GATATGATGGCAGCAATCACAG
IL-1β	F: TACCTTGCTTGTACCGAGTCG<br>R: CAGGAGGTTGTCATGTTGGTC
IL8	F: CAATGAGTCTTAGAGGTCTGGGTG<br>R: GACCTTCTTAACCCAGGGAGC

Table S2 The different protein abundances in the two media

Accession	Gene	Protein name	SG:CG
A0A2S4EU18	*fabD*	[acyl-carrier-protein] S-malonyltransferase	3.59
A0A142F8B7	*liaI*	Protein LiaI	3.36
A0A1J0F5W8	*fenA*	FenA	3.29
A0A4V7TLT2	*pks2F*	Polyketide beta-ketoacyl:ACP synthase	2.896
A0A1Y0XCW1	*pks2A*	HTH-type transcriptional regulator Pks2A	2.895
A0A0D7XBC4	*secG*	Preprotein translocase subunit SecG	2.766
A7Z7B9	*ilvC*	Ketol-acid reductoisomerase (NADP(+))	2.53
A7Z8Y5	*gapA*	Glyceraldehyde-3-phosphate dehydrogenase	2.50
A0A0D7XRH7	*aspP*	Aspartate phosphatase	2.39
A0A0D7XHS1	*prkA*	Serine protein kinase (prkA protein)	2.312
A0A268DSS9	*leuD*	3-isopropylmalate dehydratase small subunit	2.26
A0A0D7XVG2	*leuA*	2-isopropylmalate synthase	2.20
I2CAX5	*rbsK*	Ribokinase	2.14
A0A4V7TQF6	*pks2E*	[Acyl-carrier-protein] S-malonyltransferase	2.121
Q1RS57	*difA*	[Acyl-carrier-protein] S-malonyltransferase	2.095
A0A2U8RS20	*purC*	Phosphoribosylaminoimidazole-succinocarboxamide synthase	2.09
I2C8L0	*ilvH*	Acetolactate synthase small subunit	2.02
A0A142FBR9	*adeL*	Adenylosuccinate lyase	2.00
A0A142F634	*spoIIAB*	Anti-sigma F factor	1.98
A0A172XFS3	*purL*	Phosphoribosylformylglycinamidine synthase subunit	1.97
A0A2S4EQB6	*gltB*	Glutamate synthase large subunit	1.95
A0A142F735	*leuB*	3-isopropylmalate dehydrogenase	1.94
A0A142FBS7	*purH*	Bifunctional purine biosynthesis protein	1.88
A0A2U8SIN9	*zwf*	Glucose-6-phosphate 1-dehydrogenase	1.86
Q1RS47	*difD*	Acyl CoA synthetase (AMP forming)/AMP acid ligase II	1.847
A7Z7B6	*leuC*	3-isopropylmalate dehydratase large subunit	1.80
A0A4V7TNG4	*pks2C*	Malonyl CoA-acyl carrier protein transacylase	1.765
A0A0D7XV79	*purD*	Phosphoribosylamine--glycine ligase	1.72
I2CBA8	*fbaA*	Fructose-bisphosphate aldolase	1.65
Q1RS46	*difH*	Polyketide synthase type I	1.634
A0A142F8L3	*gpmI*	2,3-bisphosphoglycerate-independent phosphoglycerate mutase	1.62
A0A172XKD8	*pdk2*	Dihydrolipoamide acetyltransferase component of pyruvate dehydrogenase complex	1.61
A0A142FAQ6	*abaT*	4-aminobutyrate aminotransferase	1.60
Q1RS53	*difG*	Polyketide synthase type I	1.592
A0A268DPB8	*fabZ*	Beta-hydroxyacyl-ACP dehydratase	1.59
A0A1D9PR38	*bcaT*	Branched-chain-amino-acid aminotransferase	1.56
A0A142FBS4	*purF*	Amidophosphoribosyltransferase	1.54
A0A172XFR2	*purM*	Phosphoribosylformylglycinamidine cyclo-ligase	1.54

A0A2S4EVU1	*citS*	Citrate synthase	1.54
Q1RS45	*difI*	Polyketide synthase type I	1.511
A0A0D7XM42	*otc*	Ornithine carbamoyltransferase	0.661
A0A142F974	*spoIIID*	Stage III sporulation protein D	0.649
A0A142FCF1	*spoVR*	Stage V sporulation protein R	0.605
A0A142FAF5	*spoVT*	Stage V sporulation protein T	0.603
A0A142F629	*spoVAD*	Stage V sporulation protein AD	0.592
A0A0D7XRB9	*spoVAF*	Stage V sporulation protein AF	0.551
A0A142FA35	*ars*	Arginase	0.546
A0A2S4ER38	*spoVAE*	Stage V sporulation protein AE	0.543
A0A1Y0XME5	*sspI*	Small, acid-soluble spore protein I	0.534
A0A142FCV6	*comG*	Competence protein ComG	0.506
A0A142F9J7	*gdh*	Glutamate dehydrogenase	0.493
A0A268DNA3	*gerD*	Spore gernimation protein GerD	0.468
A0A2S4ESV9	*sigK*	RNA polymerase sigma factor	0.46
A0A2S4ES85	*acsL*	Long-chain fatty acid--CoA ligase	0.453
A0A0D7XD51	*spoVM*	Stage V sporulation protein M	0.428
I2C0G8	*sspF*	Small acid-soluble spore protein F	0.424
A0A142FAC3	*csfB*	CsfB	0.414
A0A142F9X0	*hutH*	Histidine ammonia-lyase	0.406
A0A0D7XRX3	*gerT*	Spore gernimation protein GerT	0.402
A0A142F5Z3	*mecA*	Adapter protein MecA	0.401
A0A0D7XUR3	*pckA*	Phosphoenolpyruvate carboxykinase	0.357
A0A142FAU8	*hpmO*	4-hydroxyphenylacetate 3-monooxygenase	0.336
A0A268DW19	*gerPC*	Spore gernimation protein GerPC	0.328
A0A142F569	*sspT*	Small, acid-soluble spore protein Tlp	0.32
A0A1Y0XKE5	*sspJ*	Small, acid-soluble spore protein J	0.284
A0A2S4ENA0	*hutI*	Imidazolonepropionase	0.263
A0A142F7E7	*sspA*	Small acid-soluble spore protein A	0.226
A0A2S4EMN9	*gerQ*	Spore gernimation protein GerQ	0.206
A0A2R3EMT2	*hutU*	Urocanate hydratase	0.184
